# Trends of non-melanoma skin cancer incidence in Hong Kong and projection up to 2030 based on changing demographics

**DOI:** 10.1080/07853890.2022.2154382

**Published:** 2022-12-15

**Authors:** Qingqiang Xu, Xiaoyan Wang, Yan Bai, Yan Zheng, Junbo Duan, Jianqiang Du, Xiaoming Wu

**Affiliations:** a Institute of Dermatology, Shaanxi Institution of Traditional Chinese Medicine, Shaanxi Traditional Chinese Medicine Hospital, Xi’an, Shaanxi, China; b The Key Laboratory of Biomedical Information Engineering of Ministry of Education, School of Life Science and Technology, Xi’an Jiaotong University, Xi’an, Shaanxi, China; c School of Continuing Education, Xi’an Jiaotong University, Xi’an, Shaanxi, China; d Department of Dermatology, The First Affiliated Hospital of Xi’an Jiaotong University, Xi’an, Shaanxi, China

**Keywords:** Non-melanoma skin cancer, age-period-cohort analysis, projection, decomposition

## Abstract

**Objective:**

To assess the trends in non-melanoma skin cancer (NMSC) incidence in Hong Kong from 1990 to 2019 and the associations of age, calendar period, and birth cohort, to make projections to 2030, and to examine the drivers of NMSC incidence.

**Methods:**

We assessed the age, calendar period, and birth cohort effects of NMSC incidence in Hong Kong between 1990 and 2019 using an age-period-cohort model. Using Bayesian age-period-cohort analysis with integrated nested Laplace approximations, we projected the incidence of NMSC in Hong Kong to 2030.

**Results:**

From 1990 to 2019, the age-standardized incidence rate of NMSC increased from 6.7 per 100,000 population to 8.6 per 100,000 population in men and from 5.4 per 100,000 to 5.9 per 100,000 population in women, among the 19,568 patients in the study (9812 male patients [50.14%]). The annual net drift was 2.00% (95% confidence interval [CI]: 1.50–2.50%) for men and 1.53% (95% CI: 0.95–2.11%) for women. Local drifts increased for both sexes above the 35–39-year age group. The period and cohort risk of developing NMSC tended to rise but slowed gradually in the most recent period and post-1975 birth cohort. From 2019 to 2030, it is projected that the number of newly diagnosed NMSC cases in Hong Kong will increase from 564 to 829 in men and from 517 to 863 in women. Population aging, population growth, and epidemiologic changes contributed to the increase in incident NMSCs, with population aging being the most significant contributor.

**Conclusion:**

The slowing of the period and cohort effects suggests that the rising incidence of NMSC is partly attributable to increased awareness and diagnosis. The increasing prevalence of NMSC among the elderly and an aging population will significantly impact the clinical workload associated with NMSC for the foreseeable future.

## Introduction

Non-melanoma skin cancer (NMSC) usually refers to basal cell carcinoma (BCC) and squamous cell carcinoma (SCC) [1]. While the two subtypes share many similarities, their incidence and etiology are distinct [2]. NMSC is the most common type of cancer in Caucasians, with a lifetime risk of 28–33% for BCC and 7–11% for SCC [3]. There is a clear upward trend in the incidence of NMSC worldwide, which may be attributed to an aging population and increased sun exposure [4–6]. NMSCs impose a significant economic burden on healthcare systems and can result in severe morbidity, primarily because most NMSCs occur in visible areas, such as the head, neck, and face [7]. The estimated annual cost of NMSC in the United States is $650 million, whereas Medicare spends 6–7 times that amount on melanoma treatment [8]. Nonetheless, the epidemiology of NMSC has been hampered by substantial incidence variations and the exclusion of NMSC from large cancer registries due to low mortality rates [9–11].

NMSC is not uncommon in non-Caucasians, including the Chinese [10]. According to the recent 2019 report from the Hong Kong Cancer Registry (HKCaR) [12], NMSC is the eighth (3.2% of all cancer cases) and ninth (3.0% of all cancer cases) most common cancer in men and women, respectively, in Hong Kong. Hong Kong is one of the most urbanized and westernized cities in China, has the highest life expectancy in the world, and is undergoing a more rapid transition to an aging society. Hong Kong’s population increased from 5.6 million to 7.4 million between 1991 and 2021, while the median age rose from 31.5 to 46.3 years (Census and Statistics Department, https://www.censtatd.gov.hk/en/). Population expansion and aging will inevitably increase the NMSC burden. In Hong Kong, the incidence of NMSC is low and therefore receives less attention. Although a clinic-based incidence of NMSC in Hong Kong people was reported in 2001 [13], population-based epidemiology has not been reported since. HKCaR collects data on cancer incidence and mortality throughout the territory and does not explicitly exclude NMSC, providing a unique opportunity to examine the epidemiologic trends of NMSC in Hong Kong.

This study aimed to examine the epidemiologic trends of NMSC in Hong Kong over 30 years (1990–2019) using high-quality population-based cancer registry data. Additionally, we examined the association between NMSC incidence and patient age, diagnosis period, and birth cohort. We further projected the future incidence of NMSC. Finally, we quantified the impact of demographic and epidemiological shifts on NMSC incidence trends in Hong Kong by using a decomposition algorithm to disentangle the effects of changes in population structure (population aging and growth) and epidemiological transition. Our findings put the effectiveness of relevant prevention strategies into context and contribute to better resource allocation and disease burden prediction.

## Materials and methods

### Data source

We retrieved the incidence counts of NMSC in Hong Kong from 1990 to 2019 from the HKCaR [12], based on the ICD-9 code 173 or the ICD-10 code C44. HKCaR is a population-based cancer registry in the Hong Kong Special Administrative Region of the People’s Republic of China. It is maintained according to the International Agency for Research on Cancer (IARC) of the World Health Organization. The data quality of HKCaR has achieved the highest standard for developed countries, as described by IARC. We excluded people under 20 because the incidence of NMSC in this age range is quite low. We obtained Hong Kong population estimates from the Union Nations World Population Prospects 2019 Revision (based on the UN medium-fertility variant, from 1990 to 2030) [14] by age group and sex. The data examined in this study are publicly available, do not contain personally identifiable information, and are therefore exempt from review by the Institutional Review Board.

### Statistical analyses

The study included data on the following variables: gender, year (from 1990 to 2030), age group (from 20–24 to 85+ years), age-specific incident cases of NMSC, and age-specific population. We calculated the truncated age-standardized incidence rates using the World Health Organization’s World population in 2000 as the standard. We examined the effects of age, calendar period, and birth cohorts on NMSC incidence using age-period-cohort (APC) analysis. We divided the incidence and population data into 14 five-year age groups (from 20–24 to 85+ years) and six five-year calendar periods (from 1990–1994 to 2015–2019). We calculated several APC estimators, including net drift, local drift, longitudinal age curve, period, and cohort rate ratios [15,16]. Net drift is a summary measure of the overall trend and is closely related to the estimated annual percentage changes of the age-standardized rates. Local drift is the yearly percentage change in a specific age group. The longitudinal age curve showed the age-specific rate over time adjusted for period division in the reference cohort. The period (cohort) rate ratio represents the incidence rate in a given calendar period (cohort) relative to a reference one. We chose the 1990–1994 calendar period and the 1970 birth cohort as the reference.

The APC effects were calculated utilizing the Web Tool for Age-Period-Cohort Analysis (Biostatistics Branch, National Cancer Institute, Bethesda, United States) [15]. The Wald chi-square test was used to determine the significance of the estimations. All analyses were conducted separately by sex. All statistical tests were two-sided, and a *p*-value of <0.05 was considered statistically significant.

We projected the future (2020–2030) NMSC incidence based on demographic changes using a Bayesian APC framework with integrated nested Laplace approximations [17]. The Bayesian inference treats all unknown parameters as random with appropriate prior distributions. Bayesian inference in the APC model uses second-order random walk (RW2) for smoothing priors of age, period, and cohort effects. It projects posterior incidence assuming that effects adjacent in time may be similar. RW2 is the discrete-time equivalent of a cubic smoothing spline and penalizes deviations from a linear trend. Each point of effect is predicted by linear extrapolation from its two immediate predecessors plus a random variance from a normal distribution with a zero mean. Integrated nested Laplace approximations (INLA) are utilized to approximate the posterior marginal distributions directly, thereby avoiding any MCMC sampling techniques as well as mixing and convergence concerns. The projection method has been validated elsewhere and has been shown to provide better coverage than alternative methods [17,18]. The R packages BAPC (version 0.0.34) and INLA (version 21.02.23) were used to implement the Bayesian APC model.

We attributed the changes in the number of incident NMSCs between 1990 and each year from 1991 to 2030 to three factors: population growth, population aging, and age-specific incidence rate (i.e. epidemiological changes). Epidemiological changes are variations in incident NMSCs that cannot be attributed to population growth or aging [19]. We performed the decomposition using a validated algorithm [20,21] that was unaffected by the reference year or the decomposition order. The R programming language (version 3.6.3) was used to handle and analyze the data.

## Results

**Figure 1.**
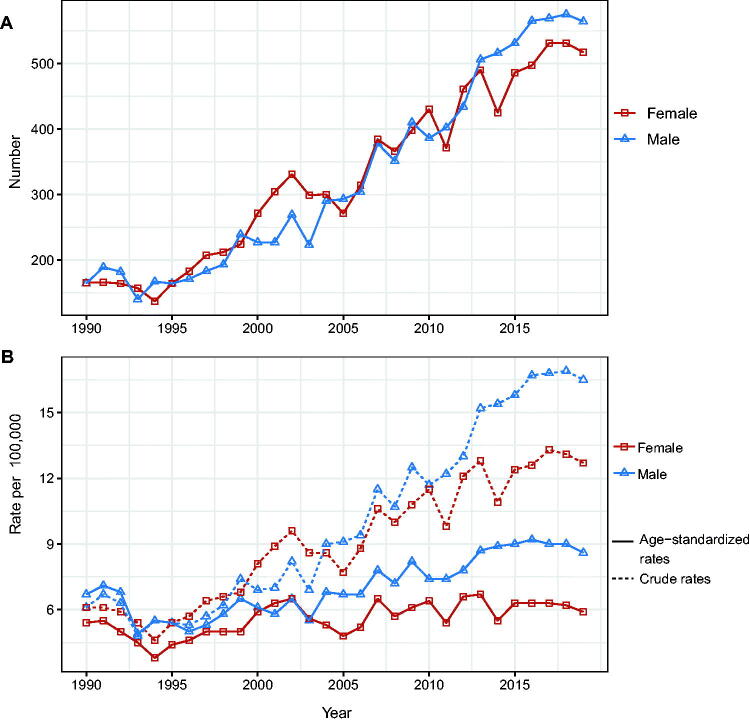
Changes in the incidence rate and the number of incident cases of NMSC in Hong Kong, 1990–2019. (A) Number of cases. (B) Age-standardized incidence rate and crude incidence rate.

A total of 19,568 patients (9812 male patients [50.14%] and 9756 female patients [49.86%]) were included in our analysis. Between 1990 and 2019, the number of men and women diagnosed with NMSC increased from 164 and 165 to 564 and 517, respectively (Figure 1(A)). The crude incidence rates for men and women grew from 6.1 per 100,000 population to 16.5 per 100,000 population and 12.7 per 100,000 population, respectively. On the other hand, the standardized incidence rate increased slightly, from 6.7 per 100,000 population and 5.4 per 100,000 to 8.6 per 100,000 and 5.9 per 100,000 for men and women, respectively (Figure 1(B)).

**Figure 2.**
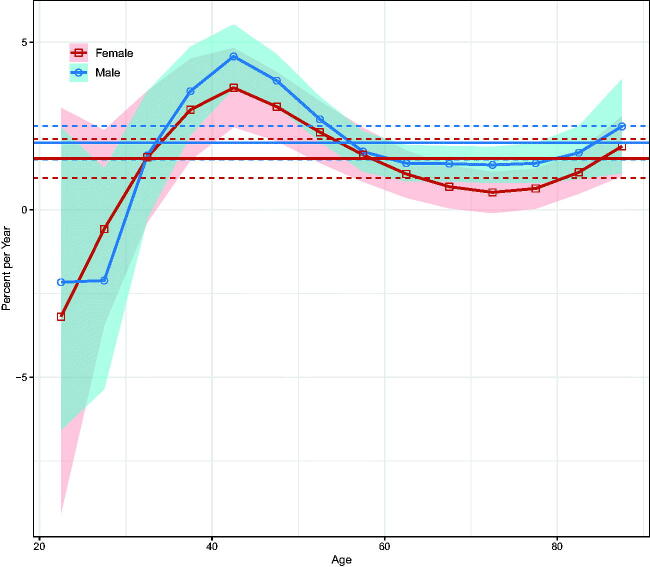
Local drifts with net drifts for NMSC incidence in Hong Kong from 1990 to 2019. Shaded areas indicate the 95% confidence interval of local drift. The horizontal lines represent the net drifts, and the horizontal dashed line corresponds to 95% confidence intervals.

Over the entire period, the net drifts indicated a statistically significant increase in NMSC incidence of 2.00% (95% confidence interval [CI]: 1.50–2.50%) per year in men and 1.53% (95% CI: 0.95–2.11%) per year in women (Figure 2). Local drifts increased for both sexes above the 35–39-year age group, with the most significant increase in the 40–45-year age group; there was some decline in younger age groups, but the 95% CIs were wide (Figure 2).

**Figure 3.**
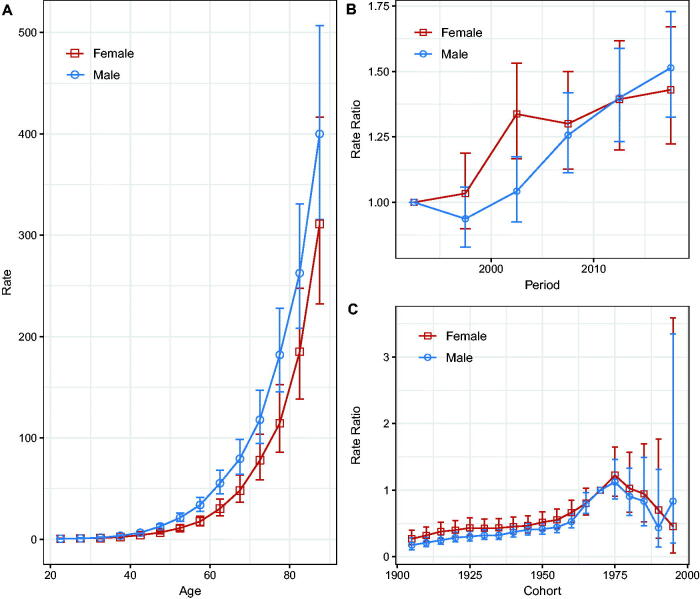
Age, calendar period, and birth cohort effects with the corresponding 95% confidence intervals on NMSC incidence rates in Hong Kong, 1990–2019. (A) Longitudinal curves of fitted age-specific rates in reference cohort adjusted for period effects; (B) rate ratios in each period relative to the reference period, adjusted for age and non-linear cohort effects; (C) rate ratios in each cohort relative to reference cohort, adjusted for age and non-linear period effects.

Compared with the reference period (1990–1994), the NMSC incidence rate in men showed an overall increasing trend, whereas, in women, the incidence rates increased until 2000–2004 and fluctuated after that. However, the 95% CIs were larger (Figure 3(A), 3(B)), suggesting that the period effect slowed down. The rate ratios of NMSC incidence for both sexes continued to increase in the pre-1975 cohort but showed a downward trend in the post-1975 birth cohort; the larger 95% CIs suggested that the cohort effect decreased (Figure 3(C)). Wald tests showed that the main estimable functions were statistically significant for both sexes (*p* < 0.05) (Table S1).

**Figure 4.**
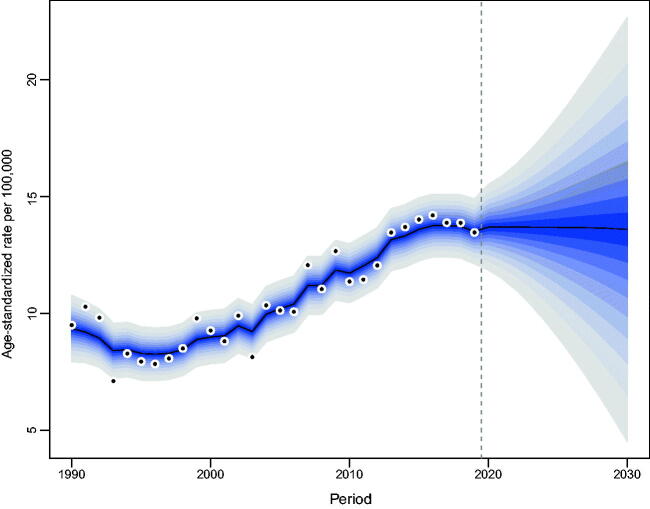
Trends and projected incidence rates for NMSC in Hong Kong men. Dots represent fitted points. Data on the right of the dashed line were projected data. Each lighter shade of blue represents an additional 10% CI.

**Figure 5.**
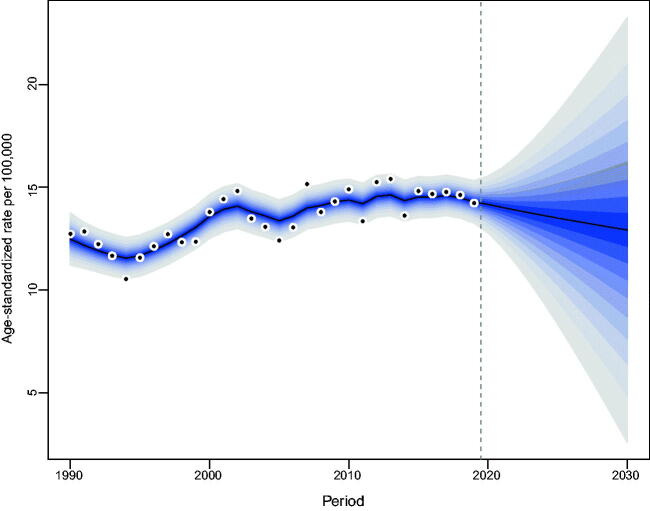
Trends and projected incidence rates for NMSC in Hong Kong women. Dots represent fitted points. Data on the right of the dashed line were projected data. Each lighter shade of blue represents an additional 10% CI.

Our projections indicated a gradual increase in the number of incident NMSCs occurring in Hong Kong. From 2019 to 2030, the number of newly diagnosed NMSC cases in Hong Kong will increase from 564 to 829 in men and from 517 to 863 in women (Tables S2, S3). The increase was most noticeable among the elderly over the age of 60. As for the age-standardized incidence rate of NMSC, men will maintain stability, while women will show a decreasing trend (Figures 4, 5).

**Figure 6.**
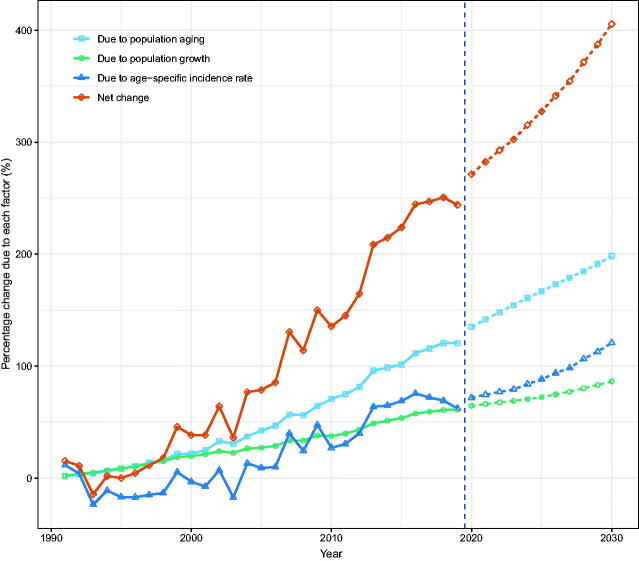
Contribution of changes in population aging, population growth, and age-specific incidence rate to changes in the number of incident NMSCs from 1991 to 2030 for Hong Kong men, using 1990 as the reference year. Data on the right of the blue dashed line was the decomposition based on the projected data.

**Figure 7.**
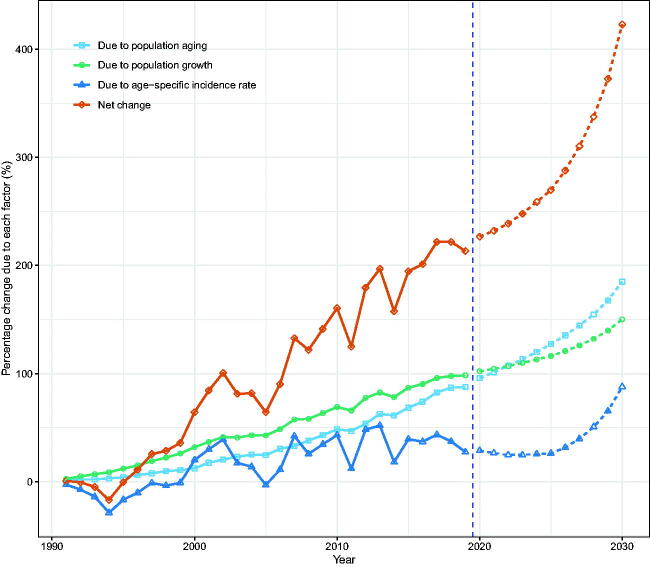
Contribution of changes in population aging, population growth, and age-specific incidence rate to changes in the number of incident NMSCs from 1991 to 2030 for Hong Kong women, using 1990 as the reference year. Data on the right of the blue dashed line was the decomposition based on the projected data.

We attributed the changed incident NMSCs to three factors: population aging, population growth, and epidemiological changes, using 1990 as the reference year. We showed that in 2019, men and women in Hong Kong experienced 400 (243.9%) and 352 (213.3%) more incident NMSCs, respectively. Among them, population aging was responsible for 198 cases (120.4%) and 144 cases (87.5%), population growth was responsible for 100 cases (61.3%) and 162 cases (98.4%), and epidemiological changes accounted for 102 cases (62.2%) and 45 cases (27.4%) (Figures 6, 7; Tables S4, S5).

According to our projections, the number of incident NMSCs in Hong Kong will continue to rise through 2030. Population aging, population growth, and epidemiologic changes contributed to the rise in incident NMSCs, with population aging contributing the most (Tables S4, S5).

## Discussion

In this study, we used APC analysis to assess the trends and reasons for the changing incidence of NMSC in Hong Kong and make projections. Both crude and age-standardized incidence rates of NMSC increased, but the latter increase was smaller, implying a role in demographic changes. We found that the period and cohort risk of developing NMSC increased but gradually slowed down in the most recent period and birth cohort. We showed that demographic factors are the main drivers of the increase in new NMSC cases in Hong Kong, with epidemiological changes also contributing. Ongoing epidemiologic surveillance and clinical studies of the disease are needed.

We quantified the effect of age, period, and cohort on the incidence of NMSC. We discovered that the period and cohort risk of developing NMSC in Hong Kong tended to increase but gradually slowed down in the most recent period and birth cohort. The main risk factor for NMSC was exposure to sunlight [2,5], with the development of BCC mainly due to intense short-term exposure. In contrast, the development of SCC was associated with cumulative sunlight exposure during adulthood [2]. However, we believe that the increased period and cohort risk of developing NMSC in the Hong Kong population may be due to a significant change in public and physician awareness of skin cancer. After all, NMSC is rarely lethal, but the site of involvement is often the head and neck, resulting in poor appearance [4,13,22]. Thus, changes in diagnostics and awareness have contributed to the observed increase in incidence, similar to what has been seen in other regions [23]. However, it isn’t easy to disentangle the effects of an increased awareness from those of increased sunlight exposure behavior.

This study contributes significantly by projecting the trend of NMSC incidence in Hong Kong. We demonstrated that the age-standardized incidence of NMSC in Hong Kong is flatter in men and decreasing in women, but the number of people developing NMSC will continue to rise. According to our population decomposition, demographic changes are the primary factor driving the increase in incident NMSCs. The increasing number and proportion of older adults have pushed more people into the high-NMSC-incidence age group. The continued growth in the number of people over 60 years of age increases the future burden of NMSC in Hong Kong.

While demographic factors account for the majority of the increased burden of NMSC, we discovered that changes in epidemiological factors also contribute to the increased burden. Because sunlight exposure is the primary epidemiological risk for NMSC, reducing sunlight exposure can help reduce the incidence of the disease. However, numerous studies have demonstrated that insufficient sun exposure has become a significant public health problem [24]. According to reports, inadequate sunlight exposure may be responsible for 340,000 deaths per year in the United States and 480,000 deaths per year in Europe [25], as well as an increased incidence of breast cancer [26], colorectal cancer [27], cardiovascular disease [28], metabolic syndrome [29], multiple sclerosis [30,31], Alzheimer’s disease [32], autism [33], and myopia [34]. Therefore, given the non-fatal nature of NMSC, Hong Kong residents should ensure that their skin is fully exposed to the sun without getting sunburned so they can get the most health benefits.

Our study has some limitations. First, because NMSC is not lethal and pigmented BCC is the most common clinical lesion in Hong Kong residents, unlike the typical rodent ulcers seen in Caucasians, many patients do not necessarily seek hospital treatment [13]. There may also be significant variation in the registration practice, suggesting that HMCaR may have underestimated the incidence of NMSC in Hong Kong. Second, the HKCaR does not distinguish between the two major subtypes of NMSC. Knowing this information would have allowed us to make the necessary adjustments to our analysis. Third, the population structure and size of Hong Kong were derived from the UN World Population Prospects, whose estimates using the medium-fertility variable may underestimate the extent of aging. Finally, because of the model complexity and longer projection periods, the prediction bias will increase and the accuracy will decrease. Some other methods may be useful to validate our findings. Despite these limitations, our findings are the best estimate of NMSC incidence, given the data available in Hong Kong.

## Conclusion

In summary, the period and cohort risk of developing NMSC in Hong Kong has gradually slowed down, but the incidence rate of NMSC in the elderly population has increased. Despite stable age-standardized incidence rates, incident NMSCs will continue to rise with Hong Kong’s aging population. More research and epidemiological evaluation of the disease are needed.

## Supplementary Material

Supplemental Material

## Data Availability

Publicly available datasets were analyzed in this study. The data can be found at Hong Kong Cancer Registry http://www3.ha.org.hk/cancereg/allages.asp/.
